# Typhoid conjugate vaccine effectiveness in Malawi: evaluation of a test-negative design using randomised, controlled clinical trial data

**DOI:** 10.1016/S2214-109X(22)00466-1

**Published:** 2022-11-25

**Authors:** Yuanyuan Liang, Amanda J Driscoll, Priyanka D Patel, Shrimati Datta, Merryn Voysey, Neil French, Leslie P Jamka, Marc Y R Henrion, Latif Ndeketa, Matthew B Laurens, Robert S Heyderman, Melita A Gordon, Kathleen M Neuzil

**Affiliations:** a Department of Epidemiology and Public Health, University of Maryland School of Medicine, Baltimore, MD, USA; b Center for Vaccine Development and Global Health, University of Maryland School of Medicine, Baltimore, MD, USA; c Malawi-Liverpool-Wellcome Programme, Kamuzu University of Health Sciences, Blantyre, Malawi; d Oxford Vaccine Group, Department of Paediatrics, University of Oxford, Oxford, UK; e Institute of Infection, Veterinary and Ecological Sciences, University of Liverpool, Liverpool, UK; f Division of Infection and Immunity, University College London, London, UK

## Abstract

**Background:**

Typhoid conjugate vaccines are being introduced in low-income and middle-income countries to prevent typhoid illness in children. Vaccine effectiveness studies assess vaccine performance after introduction. The test-negative design is a commonly used method to estimate vaccine effectiveness that has not been applied to typhoid vaccines because of concerns over blood culture insensitivity. The overall aim of the study was to evaluate the appropriateness of using a test-negative design to assess typhoid Vi polysaccharide-tetanus toxoid conjugate vaccine (Vi-TT) effectiveness using a gold standard randomised controlled trial database.

**Methods:**

Using blood culture data from a randomised controlled trial of Vi-TT in Malawi, we simulated a test-negative design to derive vaccine effectiveness estimates using three different approaches and compared these to randomised trial efficacy results. In the randomised trial, 27 882 children aged 9 months to 12 years were randomly assigned (1:1) to receive a single dose of Vi-TT or meningococcal capsular group A conjugate vaccine between Feb 21 and Sept 27, 2018, and were followed up for blood culture-confirmed typhoid fever until Sept 30, 2021.

**Findings:**

For all three test-negative design approaches, vaccine effectiveness estimates (test-negative design A, 80·3% [95% CI 66·2 to 88·5] *vs* test-negative design B, 80·5% [66·5 to 88·6] *vs* test-negative design C, 80·4% [66·9 to 88·4]) were almost identical to the randomised trial results (80·4% [95% CI 66·4 to 88·5]). Receipt of Vi-TT did not affect the risk of non-typhoid fever (vaccine efficacy against non-typhoid fever –0·4% [95% CI –4·9 to 3·9] *vs* –1% [–5·6 to 3·3] *vs* –2·5% [–6·4 to 1·3] for test-negative design A, test-negative design B, and test-negative design C, respectively).

**Interpretation:**

This study validates the test-negative design core assumption for typhoid vaccine effectiveness estimation and shows the accuracy and precision of the estimates compared with the randomised controlled trial. These results show that the test-negative design is suitable for assessing typhoid conjugate vaccine effectiveness in post-introduction studies using blood culture surveillance.

**Funding:**

Bill & Melinda Gates Foundation.

## Introduction

Typhoid fever, a systemic infection caused by *Salmonella enterica* serovar Typhi (*S* Typhi), is transmitted through contaminated food and water. In 2019, there were an estimated 9·24 million cases of typhoid fever globally and 110 000 deaths, with the greatest burden in low-income and middle-income countries (LMICs) in sub-Saharan Africa and Asia.^1^ Increasing antimicrobial resistance, including the emergence of extensively drug resistant isolates, limits treatment options in many settings and has created an urgency to adopt effective preventive measures.^2^ In 2017, WHO recommended routine use of typhoid conjugate vaccine in children aged 6 months and older in endemic settings.^3^ Since then, excellent typhoid conjugate vaccine efficacy has been shown in randomised controlled trials in LMICs.^4^, ^5^, ^6^, ^7^ With the support of Gavi, the Vaccine Alliance, typhoid conjugate vaccine has been introduced into routine immunisation programmes in Pakistan, Liberia, Zimbabwe, and Nepal, with more countries expected to introduce it soon.^8^ In 2022, the WHO Strategic Advisory Group of Experts on Immunization reviewed newly available data from surveillance studies, efficacy trials, and early country introductions and reaffirmed the typhoid conjugate vaccine recommendations.^9^

Post-introduction vaccine evaluations are necessary to understand vaccine performance under real-world conditions and to provide information to sustain vaccination programmes and support decision making. Case-control studies offer an efficient approach to estimate vaccine effectiveness, because cases are identified based on disease status (ie, the outcome of interest), and sample size requirements tend to be smaller than for other study designs, especially for diseases with low prevalence.^10^ However, identification of appropriate controls can be challenging.^11^ For example, controls recruited from the community might be less likely to access health care when they are ill and less likely to be immunised, compared with cases recruited at health facilities. This confounding can lead to biased estimates of vaccine effectiveness. A design that reduces potential bias due to differential health-care-seeking behaviours is the test-negative design.^12^ The test-negative design is a variation of the case-control study in which participants who present with illness are classified as cases or controls on the basis of diagnostic testing for the pathogen in question.^13^

**Research in context**

**Evidence before this study**

As typhoid conjugate vaccines are introduced into national immunisation programmes, post-introduction monitoring of vaccine performance is important to understand how vaccines work under real-world conditions. The test-negative design has previously been efficiently used to evaluate post-introduction vaccine effectiveness for other vaccines. We aimed to validate the usefulness of the test-negative design for typhoid conjugate vaccines using a gold-standard randomised controlled trial database. We searched PubMed on Oct 17, 2022, with no date or language restrictions, using the terms (typhoid conjugate vaccine) AND (randomised controlled trial). Our search identified 47 papers, including four randomised controlled trials investigating typhoid conjugate vaccines, and no studies reporting the use of the randomised trials for test-negative design validation in relation to typhoid conjugate vaccines. We did a second search of PubMed on Oct 17, 2022, with no date or language restrictions, using the terms (typhoid conjugate vaccine) AND (case control study or test negative design). This second search identified 20 papers, and did not find any studies reporting the use of the randomised trials for test-negative design validation in relation to typhoid conjugate vaccines.

**Added value of this study**

To our knowledge, this is the first study to rigorously evaluate the use of a test-negative design to estimate vaccine effectiveness. This study validated the test-negative design core assumption that typhoid conjugate vaccine has no effect on febrile illnesses that are not caused by typhoid. Furthermore, this study showed the accuracy and precision of vaccine effectiveness estimates derived using the test-negative design compared with gold-standard randomised controlled trial vaccine efficacy results in a Malawian paediatric population. Importantly, the effect of blood culture sensitivity was minimal for vaccine effectiveness estimation accuracy, especially when typhoid-positive cultures comprised fewer than 10% of all blood cultures.

**Implications of all the available evidence**

The effectiveness of new vaccines should be evaluated after their introduction by use of the most rigorous approach possible. In the absence of randomised trials, epidemiological designs can be used to estimate vaccine effectiveness. Our study provides evidence to support the use of the test-negative design to measure post-introduction vaccine effectiveness of typhoid conjugate vaccines in Malawi and other similar settings. This efficient method to evaluate the performance of newly introduced typhoid conjugate vaccines could be a potential tool to generate crucial evidence for the continued support of existing vaccination programmes and to support decisions on whether to introduce typhoid conjugate vaccines in other countries. As in all observational study designs, the test-negative design remains susceptible to selection bias, confounding, and misclassification of vaccine and outcome status. The test-negative design should be considered only when a case-control study design is appropriate.

The test-negative design has been extensively used to measure pneumococcal, influenza, rotavirus, and COVID-19 vaccine effectiveness.^14^, ^15^, ^16^ As an efficient and simple study design, the test-negative design offers advantages for typhoid conjugate vaccine evaluation after introduction in LMICs. However, not all diseases are amenable to the test-negative design. For typhoid, the validity of the test-negative design might be affected by low disease incidence and reliance on moderately sensitive blood culture for diagnosis.^17^

In this study, we evaluate the test-negative design to estimate typhoid conjugate vaccine effectiveness using a previously published randomised trial of typhoid conjugate vaccine (typhoid Vi polysaccharide-tetanus toxoid conjugate vaccine [Vi-TT]) in Malawi as the gold standard comparator.^6^ The objectives of this study were threefold: to verify the core assumption of the test-negative design, that Vi-TT has no effect on non-typhoid fever, to compare vaccine effectiveness derived by the test-negative design with randomised trial efficacy results, and to assess the effect of case-control matching, vaccine miscalculation, and blood culture sensitivity to detect typhoid fever on vaccine effectiveness estimation in the test-negative design.

## Methods

### Study design and participants

Participant eligibility, enrolment, and population definitions in the comparator randomised trial have been described previously.^6^, ^18^ This parent trial was approved by the Malawi National Health Sciences Research Committee; the Malawi Pharmacy, Medicines, and Regulatory Authority; the institutional review board at the University of Maryland, Baltimore (MD, USA); and the research ethics committee at the University of Liverpool (Liverpool, UK). The present study was a post-hoc analysis of existing data and is detailed in the original study statistical analysis plan.

Briefly, in the randomised trial per-protocol sample, 27 882 Malawian children in Blantyre aged 9 months to 12 years were randomly assigned (1:1) to receive a single dose of Vi-TT or meningococcal capsular group A conjugate vaccine (MenA) from Feb 21 to Sept 27, 2018. Participants (13 945 in the Vi-TT group and 13 937 in the MenA group; median age at vaccination 6 years; 14 198 [51%] of 27 882 participants were female) were followed up for blood culture-confirmed typhoid fever with passive surveillance. Participants who presented with febrile illness at outpatient health centres and tertiary facilities in two urban townships (Ndirande and Zingwangwa) in Blantyre, Malawi had a blood culture sample collected if they met one of the following protocol-defined specimen collection criteria: subjective fever for at least 72 h, measured fever of at least 38°C, or hospitalisation with history of fever of any duration. Blood culture results were classified as follows: positive for typhoid, negative for typhoid but positive for a non-typhoid pathogen, or negative for any pathogen or positive for contaminants. All facilities within the study area with blood culture capacity were included in the randomised trial. Vaccine efficacy results for participants followed up until April 3, 2020 (ie, 18–24 months after vaccination), have been reported previously.^6^ This evaluation used efficacy data from participants followed up until Sept 30, 2021. The test-negative design sample included all participants in the randomised trial who had a blood culture sample collected at least 14 days after vaccination with a reported blood culture result. Blood cultures that grew *S* Typhi were classified as test-positive. Negative blood cultures, and those that grew a non-typhoid pathogen or a contaminant, were considered test-negative.

In the test-negative design, only participants with at least one blood culture specimen collected were considered for the vaccine effectiveness analysis. Because some participants had more than one episode with a blood culture specimen collected, three test-negative design samples were selected, corresponding to three different analysis approaches to estimate the vaccine effectiveness, as described previously, as follows: participant-based analysis sample without censoring for typhoid (test-negative design A); participant-based analysis sample with censoring for typhoid (test-negative design B); and specimen-based analysis sample (test-negative design C).^19^ Both test-negative design A and test-negative design B are participant-based analysis samples where cases were participants who had ever had a typhoid-positive blood culture test during the study period. In test-negative design A, controls included participants with an episode of non-typhoid illness, without excluding those who might have tested positive for typhoid at another time within the study period. In test-negative design B, controls included only participants with an episode of non-typhoid illness, who never had a positive test for typhoid at any time during the study period. In test-negative design C, the units of analysis were specimens rather than individuals, and cases were typhoid-positive specimens and controls were typhoid-negative specimens.

### Statistical analysis

In the randomised controlled trial per-protocol analysis, vaccine efficacy against blood culture-confirmed typhoid fever up to Sept 30, 2021, was calculated as (1 – incidence rate ratio [IRR]) × 100%, where IRR is the ratio of the incidence rate in the Vi-TT group to that in the MenA group, as described in the study protocol and the published 18–24-month analysis.^6^, ^18^ In a sensitivity analysis, vaccine efficacy was also calculated as (1– relative risk [RR]) × 100%, where RR is the relative risk of blood culture-confirmed typhoid fever in the Vi-TT group compared with the MenA group.

In each of the three test-negative design samples, vaccine effectiveness against typhoid fever was calculated as (1 – odds ratio [OR]) × 100%, where OR is the relative odds of Vi-TT vaccination in cases compared with controls. The Woolf approximation method was used to compute 95% CIs for the ORs. All randomised trial participants were used to test the core assumption of the test-negative design, that Vi-TT has no effect on non-typhoid fever. Vaccine efficacy against non-typhoid illness was calculated as (1 – RR) × 100%, where RR is the relative risk of participants or specimens testing negative for typhoid in the Vi-TT group compared with the MenA group. Three methods were used (participant-based with or without censoring and specimen-based analysis).^19^ The exact method was used to compute 95% CIs for the RRs. All vaccine effectiveness analyses described against typhoid and non-typhoid fever were repeated for each of the following prespecified subgroups: age (<5 years *vs* ≥5 years), sex (male *vs* female), and study site.

Instead of using all available controls, a matched test-negative design was simulated to examine the effect of matching key study variables on vaccine efficacy estimates. A matched test-negative design is preferable because it improves the comparability between cases and controls and requires data collection on fewer individuals. Mahalanobis multivariate-distance nearest-neighbour matching without replacement was used to match each typhoid-positive specimen with three typhoid-negative specimens (appendix pp 1–2). Cases and controls were matched exactly on age categories (<2 years *vs* 2 years to <5 years *vs* ≥5 years) and study site. To reduce potential bias due to typhoid seasonality, blood culture dates were matched within 20 days for each case-control pair (295 [97%] of 303 specimens were matched within 7 days). Vaccine effectiveness against typhoid fever was calculated as (1 – OR) × 100%, where OR was estimated using mixed-effects logistic regression to account for the potential correlations among matched cases and controls introduced by the study design.

To examine the effect of vaccine misclassification on test-negative design vaccine effectiveness estimation, we assumed that poor vaccination records affected both cases and controls equally. Different probabilities of misclassifying vaccinated individuals as unvaccinated and misclassifying unvaccinated individuals as vaccinated were considered (appendix pp 3–4). The estimated vaccine effectiveness with corresponding 95% CIs under various overall vaccine misclassification rates was calculated. The test-negative design specimen-based method was used with vaccine effectiveness calculated as (1 – OR) × 100%.

To examine the effect of blood culture sensitivity in detecting typhoid fever on vaccine effectiveness estimation, the test-negative design specimen-based method was used.^19^ Fixed blood culture sensitivity was assumed, that is, sensitivity does not depend on time since infection or vary across individuals. The Vi-TT vaccination rate among the false-negative specimens was assumed to be the same as the vaccination rate among the typhoid-positive specimens. Additionally, we assumed no misclassification of vaccination status. Various blood culture positivity rates (ie, the proportion of blood culture specimens that were typhoid-positive) were also used to examine the effect of typhoid prevalence on vaccine effectiveness estimation using a test-negative design (appendix p 5).

A data and safety management board oversaw the randomised controlled trial, but not the present study. All analyses were done using Stata/SE version 17.

### Role of the funding source

The funder of the study had no role in study design, data collection, data analysis, data interpretation, or writing of the report.

## Results

**Figure 1:**
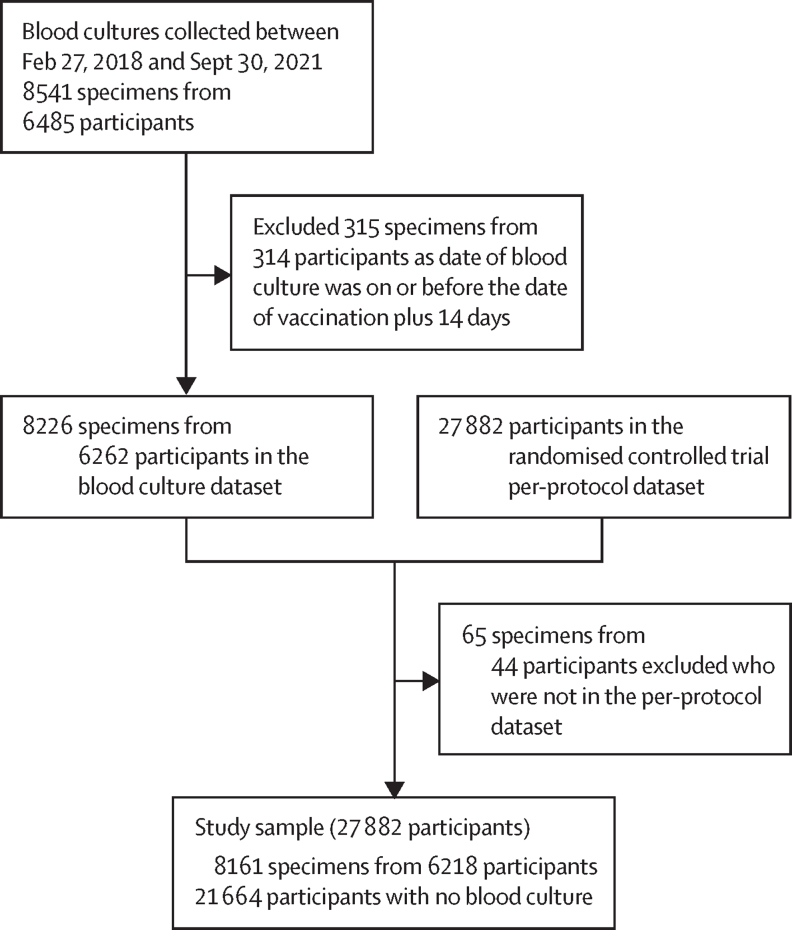
Study sample derivation

The study sample derivation is shown in figure 1. After excluding those who did not meet per-protocol criteria, the final study sample included 27 882 unique children, of whom 21 664 had no blood culture specimen collected (non-test-negative design sample) and 6218 had at least one blood culture specimen (test-negative design sample). Among the 6218 children in the test-negative design sample, 4759 (76·5%) had only one blood culture specimen collected, 1123 (18·1%) had two blood culture specimens collected, and 336 (5·4%) had three or more blood culture specimens collected, comprising a total of 8161 blood culture specimens. There were 101 typhoid-positive specimens (cases) from 97 unique children and 8060 typhoid-negative specimens (controls) from 6121 unique children.

Compared with children in the non-test-negative design sample (table 1), children who had at least one blood culture specimen collected and hence were included in the test-negative design sample were significantly younger, with a higher proportion enrolled from the Zingwangwa study site. Sex and vaccine group distributions did not differ between the test-negative design sample and the non-test-negative design sample.

**Table 1:** Participant baseline characteristics by availability of blood culture specimens

		**Without blood culture specimens, non-test-negative design sample (n=21 664)**	**With blood culture specimens, test-negative design sample (n=6218)**	**Total, randomised controlled trial per-protocol sample (n=27 882)**	**p value**
Vaccine group					0·64
	MenA	10 812 (49·9%)	3125 (50·3%)	13 937 (50·0%)	..
	Vi-TT	10 852 (50·1%)	3093 (49·7%)	13 945 (50·0%)	..
Sex					0·95
	Male	10 635 (49·1%)	3049 (49·0%)	13 684 (49·1%)	..
	Female	11 029 (50·9%)	3169 (51·0%)	14 198 (50·9%)	..
Age, years					<0·0001
	Mean (SD)	6·5 (3·2)	4·7 (3·1)	6·1 (3·3)	..
	Median (IQR)	7 (4–9)	4 (2–7)	6 (3–9)	..
	Range	0·8–12·0	0·8–12·0	0·8–12·0	..
Age categories, years					<0·0001
	<5	6759 (31·2%)	3443 (55·4%)	10 202 (36·6%)	..
	≥5	14 905 (68·8%)	2775 (44·6%)	17 680 (63·4%)	..
Study site					<0·0001
	Ndirande	13 771 (63·6%)	3739 (60·1%)	17 510 (62·8%)	..
	Zingwangwa	7893 (36·4%)	2479 (39·9%)	10 372 (37·2%)	..

Data are n (%), unless otherwise indicated. MenA=meningococcal capsular group A conjugate vaccine. Vi-TT=typhoid Vi polysaccharide-tetanus toxoid-conjugate vaccine.

**Figure 2:**
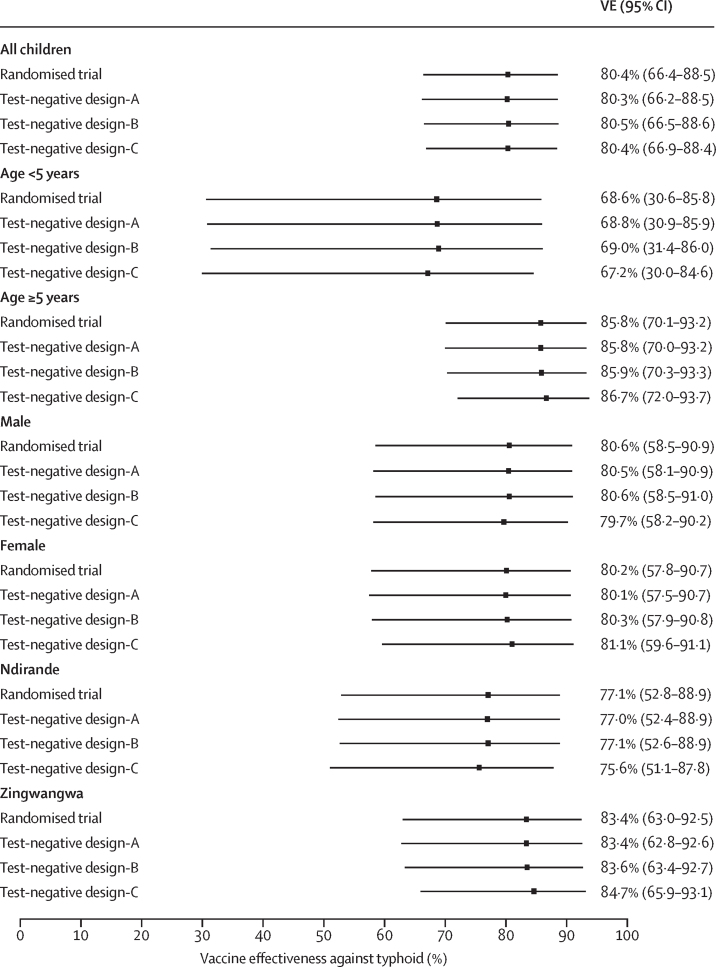
Effect of vaccine on blood culture-confirmed typhoid overall and by subgroups VE=Vaccine efficacy in the randomised controlled trial or vaccine effectiveness in the test-negative design.

Vaccine efficacy against typhoid fever in the randomised trial up to September, 2021 (ie, at least 3 years after vaccination), was 80·4% (95% CI 66·4 to 88·5) based on the IRR method and 80·3% (66·3 to 88·4) based on the RR method. For all three test-negative design approaches, vaccine effectiveness estimates (test-negative design A, 80·3% [95% CI 66·2 to 88·5] *vs* test-negative design B, 80·5% [66·5 to 88·6] *vs* test-negative design C, 80·4% [66·9 to 88·4]) were almost identical to the randomised trial results (table 2, figure 2). Receipt of Vi-TT did not affect the risk of non-typhoid fever (vaccine efficacy against non-typhoid fever –0·4% [95% CI –4·9 to 3·9] *vs* –1% [–5·6 to 3·3] *vs* –2·5% [–6·4 to 1·3] for test-negative design A, test-negative design B, and test-negative design C, respectively; table 2).

**Table 2:** Influence of vaccine against blood culture-confirmed typhoid and non-typhoid illnesses, estimated by per-protocol and three test-negative design analysis approaches

	**Participant-based analysis**					Specimen-based analysis*	
	Total	Test-positive for typhoid	Test-negative with no censoring†	Test-negative with censoring‡	No blood culture	Test-positive specimens	Test-negative specimens
Vi-TT	13 945	16/97 (16·5%)	3087/6166 (50·1%)	3077/6121 (50·3%)	10 852	17/101 (16·8%)	4092/8060 (50·8%)
MenA	13 937	81/97 (83·5%)	3079/6166 (49·9%)	3044/6121 (49·7%)	10 812	84/101 (83·2%)	3968/8060 (49·2%)
VE against typhoid§ (95% CI), p value	..	80·4% (66·4 to 88·5)¶, p<0·0001	80·3% (66·2 to 88·5)‖, p<0·0001	80·5% (66·5 to 88·6)**, p<0·0001	..	..	80·4% (66·9 to 88·4)††, p<0·0001
VE against non-typhoid‡‡ (95% CI), p value	..	..	−0·4% (−4·9 to 3·9)‖, p=0·87	−1·0% (−5·6 to 3·3)**, p=0·65	..	..	−2·5% (−6·4 to 1·3)††, p=0·20

Data are n or n/N (%), unless otherwise indicated. MenA=meningococcal capsular group A conjugate vaccine. VE=vaccine efficacy in the randomised controlled trial or vaccine effectiveness in the test-negative design. Vi-TT=typhoid Vi polysaccharide-tetanus toxoid-conjugate vaccine.

* Cases are typhoid-positive specimens and controls are typhoid-negative specimens.

† Controls include participants with an episode of non-typhoid illness, without censoring for typhoid (ie, controls might have tested positive for typhoid at another timepoint).

‡ Controls include participants with an episode of non-typhoid illness, with censoring for typhoid (ie, controls exclude participants who ever had a test that was typhoid-positive during the study period).

§ VE=(1 – odds ratio) × 100%, using the test-negative design sample only.

¶ VE=(1 – incidence rate ratio) × 100%.

‖ Test-negative design A.

** Test-negative design B.

†† Test-negative design C.

‡‡ VE=(1 – risk ratio) × 100%, using the whole randomised controlled trial.

Subgroup analyses were done by age, sex, and study site (figure 2; appendix pp 6–7). All three test-negative design analysis approaches produced similar results, with point estimates and corresponding 95% CIs almost identical to the per-protocol randomised trial results (figure 2). Additionally, Vi-TT had no effect on non-typhoid fever in all subgroups (appendix pp 6–7).

The simulated 1:3 case-control matched test-negative design (101 typhoid-positive specimens *vs* 303 typhoid-negative specimens) produced a vaccine effectiveness estimate of 80·9% (95% CI 66·4–89·2; appendix p 8). These results were almost identical to the test-negative design, with all 8060 controls (80·4% [66·9–88·4]) and to the original randomised trial results (vaccine efficacy against typhoid fever: (1 – IRR) × 100%=80·4% [66·4–88·5] or (1 – RR) × 100%=80·3% [66·3–88·4]).

Vaccine misclassification might have affected the estimation of vaccine effectiveness in a test-negative design (table 3). If the classification of vaccination status was 100% correct (ie, misclassification rate 0%—gold standard), the estimated vaccine effectiveness against typhoid was 80·4% (95% CI 66·9–88·4). If the overall misclassification rate was 5%, the estimated vaccine effectiveness was 84·0% (70·8–91·3) when misclassification occurred in one direction only (misclassifying vaccinated as unvaccinated, eg, due to the loss of vaccination cards) for both cases and controls; the lowest possible vaccine effectiveness was 67·0% (47·0–79·5) and the highest possible was 89·3% (80·4–94·2) when differential misclassification was allowed (appendix pp 3–4). Both the lowest and the highest vaccine effectiveness estimate 95% CIs overlapped with the 95% CI for the gold standard vaccine effectiveness estimate, suggesting the differences were not statistically significant. However, when the overall misclassification rate was 10% or higher, the lowest possible vaccine effectiveness and the highest possible vaccine effectiveness were significantly different from the gold standard vaccine effectiveness estimate, suggesting the estimates under these scenarios were no longer reliable or consistent given the accurate vaccination status (table 3).

**Table 3:** Effect of overall vaccine misclassification rate (p_1_ + p_2_) on vaccine effectiveness estimation by test-negative design specimen-based analysis

		**Cases vaccinated by Vi-TT**	**Controls vaccinated by Vi-TT**	VE against typhoid*(95% CI)
0% vaccine misclassification—gold standard		17/101 (16·8%)	4092/8060 (50·8%)	80·4% (66·9 to 88·4)
5% vaccine misclassification				
	Misclassifying vaccinated as unvaccinated, both groups†	12/101 (11·9%)	3689/8060 (45·8%)	84·0% (70·8 to 91·3)
	Differential misclassification, lowest possible VE‡	22/101 (21·8%)	3689/8060 (45·8%)	67·0% (47·0 to 79·5)
	Differential misclassification, highest possible VE§	12/101 (11·9%)	4495/8060 (55·8%)	89·3% (80·4 to 94·2)
10% vaccine misclassification				
	Misclassifying vaccinated as unvaccinated, both groups†	7/101 (6·9%)	3286/8060 (40·8%)	89·2% (76·7 to 95·0)
	Differential misclassification, lowest possible VE‡	27/101 (26·7%)	3286/8060 (40·8%)	47·0% (17·5 to 66·0)
	Differential misclassification, highest possible VE§	7/101 (6·9%)	4898/8060 (60·8%)	95·2% (89·6 to 97·8)
15% vaccine misclassification				
	Misclassifying vaccinated as unvaccinated, both groups†	2/101 (2·0%)	2883/8060 (35·8%)	96·4% (85·3 to 99·1)
	Differential misclassification, lowest possible VE‡	32/101 (31·7%)	2883/8060 (35·8%)	16·9% (−27·0 to 45·4)
	Differential misclassification, highest possible VE§	2/101 (2·0%)	5301/8060 (65·8%)	98·9% (95·7 to 99·7)
20% vaccine misclassification				
	Misclassifying vaccinated as unvaccinated, both groups†	0	2480/8060 (30·8%)	100% (91·4 to 100)
	Differential misclassification, lowest possible VE‡	37/101 (36·6%)	2480/8060 (30·8%)	−30·1% (−95·5 to 13·5)
	Differential misclassification, highest possible VE§	0	5704/8060 (70·8%)	100% (98·4 to 100%)

Data are n/N (%), unless otherwise indicated. See the appendix (pp 3–4) for further details. p_1_=probability of misclassifying vaccinated as unvaccinated. p_2_=probability of misclassifying unvaccinated as vaccinated. VE=vaccine effectiveness. Vi-TT=typhoid Vi polysaccharide-tetanus toxoid-conjugate vaccine.

* VE=(1 – odds ratio) × 100%.

† Only misclassifying vaccinated as unvaccinated for both cases and controls due to the loss of vaccination cards, that is, p_1_ + p_2_=p_1_, hence p_2_=0 among both groups.

‡ p_1_=0 among cases (misclassifying unvaccinated as vaccinated among cases) and p_2_=0 among controls (misclassifying vaccinated as unvaccinated among controls), resulting in the lowest possible VE.

§ p_2_=0 among cases (misclassifying vaccinated as unvaccinated among cases) and p_1_=0 among controls (misclassifying unvaccinated as vaccinated among controls), resulting in the highest possible VE.

101 (1·2%) of 8161 blood culture specimens were positive for *S* Typhi. As blood culture sensitivity decreased from 100% to 30%, the adjusted point estimates of vaccine effectiveness increased only slightly, from 80·4% to 81·%, but the width of the 95% CIs became much narrower, decreasing from 0·215 to 0·111 (table 4; appendix p 5). Additionally, as the proportion of blood cultures that were typhoid-positive increased from 1·2% to 10·0%, the effect of blood culture sensitivity on the point estimate of vaccine effectiveness became stronger, although still not significantly different, as shown by overlapping 95% CIs in all scenarios except the scenario in which blood culture sensitivity was 30% and 10% of blood cultures were positive for *S* Typhi (table 4).

**Table 4:** Effect of blood culture test sensitivity on vaccine effectiveness estimation by test-negative design specimen-based analysis, stratified by blood culture positivity rate

	Adjusted blood culture typhoid-positive*	Adjusted cases vaccinated*	Adjusted controls vaccinated*	Adjusted VE against typhoid*†**(95% CI)**
**Observed blood culture typhoid positivity 101/8161 (1·2%)**				
100% BCS	101/8161 (1·2%)	17/101 (16·8%)	4092/8060 (50·8%)	80·4% (66·9–88·4)
80% BCS	126/8161 (1·5%)	21/126 (16·7%)	4088/8035 (50·9%)	80·7% (69·1–87·9)
50% BCS	202/8161 (2·5%)	34/202 (16·8%)	4075/7959 (51·2%)	80·7% (72·0–86·7)
30% BCS	337/8161 (4·1%)	57/337 (16·9%)	4052/7824 (51·8%)	81·0% (74·7–85·8)
**Observed blood culture typhoid positivity 408/8161 (5·0%)**				
100% BCS	408/8161 (5·0%)	69/408 (16·9%)	4040/7753 (52·1%)	81·3% (75·7–85·6)
80% BCS	510/8161 (6·2%)	86/510 (16·9%)	4023/7651 (52·6%)	81·7% (76·8–85·6)
50% BCS	816/8161 (10·0%)	137/816 (16·8%)	3972/7345 (54·1%)	82·9% (79·3–85·8)
30% BCS	1360/8161 (16·7%)	228/1360 (16·8%)	3881/6801 (57·1%)	84·8% (82·4–87·0)
**Observed blood culture typhoid positivity 816/8161 (10·0%)**				
100% BCS	816/8161 (10·0%)	137/816 (16·8%)	3972/7345 (54·1%)	82·9% (79·3–85·8)
80% BCS	1020/8161 (12·5%)	171/1020 (16·8%)	3938/7141 (55·1%)	83·6% (80·6–86·2)
50% BCS	1632/8161 (20·0%)	274/1632 (16·8%)	3835/6529 (58·7%)	85·8% (83·7–87·7)
30% BCS	2720/8161 (33·3%)	457/2720 (16·8%)	3652/5441 (67·1%)	90·1% (88·9–91·2)

Data are n/N (%), unless otherwise indicated. We assumed the vaccination rate among the false negatives was the same as the vaccination rate among the cases (appendix p 5). BCS=blood culture sensitivity. VE=vaccine effectiveness.

* Adjusted by blood culture sensitivity.

† VE=(1 – odds ratio) × 100%.

## Discussion

In a paediatric population in Malawi, estimates of Vi-TT effectiveness against blood culture-confirmed typhoid fever derived by the simulated test-negative design were almost identical to the gold standard randomised controlled trial vaccine efficacy results. Vi-TT had no effect on non-typhoid fever, further validating a core assumption of the test-negative design. In subgroup analyses by age, sex, and study site, the results remained highly consistent with the randomised trial vaccine efficacy estimates, and Vi-TT similarly had no effect on non-typhoid fever. Further evidence of test-negative design accuracy was shown in a simulated 1:3 case-control matched test-negative design analysis, which produced almost identical results to test-negative design analyses that used all controls, and to the original randomised trial. The finding that case-control matching made little difference to efficacy estimates is consistent with work by Dean and colleagues.^20^ Our results support use of a matched test-negative design study as a suitable method to estimate typhoid conjugate vaccine effectiveness in preventing blood culture-confirmed *S* Typhi as these vaccines are introduced in low-resource settings.

Although the test-negative design offers strong advantages and is commonly used to measure the effectiveness of influenza, rotavirus, COVID-19, and other vaccines,^14^, ^21^, ^22^, ^23^ this study design remains susceptible to the biases present in all observational studies. These biases include selection bias, confounding, and misclassification of vaccine and outcome status. A concern specific to the test-negative design is that because cases and test-negative controls are selected among only those who present to health facilities with illness, there is a potential for the study sample to misrepresent the broader population.^24^ This concern could be mitigated when the outcome is severe illness that is more likely to result in care-seeking. Knowledge of the care-seeking behaviours of the population of interest is important when interpreting vaccine effectiveness estimates derived by the test-negative design and when considering the appropriateness of this design for a specific setting. A strength of our study is the use of data from a randomised controlled trial of Vi-TT in Malawi.^6^ Unlike many clinical trials that restrict eligibility for enrolment and hence might not be representative of the broader population, this randomised trial had few exclusion criteria and was designed to characterise the general population of Malawian children aged 9 months to 12 years in the Blantyre area.^18^ Additionally, although the test-negative design sample was significantly younger and more likely to be enrolled from the Zingwangwa site than the non-test-negative design sample, the accuracy and precision of the vaccine effectiveness estimates were nearly identical to the classic randomised trial vaccine efficacy results. Therefore, the issues of both observable and unobservable confounding were less of a concern in this study.

Outcome misclassification due to imperfect accuracy of the diagnostic test has been identified as a concern for the test-negative design, with low test specificity posing a greater threat to validity than low test sensitivity.^25^, ^26^ Although blood cultures are highly specific, the diagnostic sensitivity of blood culture for *S* Typhi is estimated to be 50–75% across various settings and might be reduced by suboptimal blood volumes and previous antimicrobial use.^17^ In our sensitivity analyses, the effect of blood culture sensitivity was trivial for vaccine effectiveness estimation accuracy (ie, point estimation), when *S* Typhi-positive cultures comprised less than 10% of all blood cultures. In scenarios where *S* Typhi-positive cultures made up 10% or more of all blood cultures, vaccine effectiveness was underestimated if no adjustments were made for low blood culture sensitivity. In a multisite surveillance study done between November, 2016, and December, 2018, the proportion of *S* Typhi-positive blood cultures ranged from 3% in Malawi to 6% in Nepal and Bangladesh.^27^ In these settings, our results indicate that vaccine effectiveness estimation by test-negative design would remain accurate. However, during an outbreak in Pakistan, 775 (31%) of 2469 blood culture specimens collected from children in a cohort study grew *S* Typhi.^28^ In this scenario, the test-negative design might not be an appropriate choice for vaccine effectiveness estimation and was not the method used in the study in Pakistan. Additionally, blood culture sensitivity significantly affected the precision of vaccine effectiveness estimation (ie, the width of the 95% CIs). Therefore, more participants are required to achieve the same power due to false negatives (ie, cases misclassified as controls) and larger variance. A limitation of our study is that the results were based on the fixed blood culture sensitivity assumption. However, blood culture sensitivity might vary with time since infection or other factors, such as blood collection volume or previous antibiotic use. Additional data and methodologies are needed to adjust for time-dependent and subject-dependent blood culture sensitivity.

Unlike randomised trials, in which vaccination history is known for each participant, misclassification of vaccination status is a concern for any observational study design, especially in settings where vaccine records are not electronic or centralised. In our sensitivity analysis, if the overall misclassification of vaccination status was 10% or higher, the vaccine effectiveness estimate was no longer reliable; this confirms that the accuracy of vaccination status is crucial in a test-negative design and supports efforts to improve the recording and retention of vaccination data at the individual level, in national programmes and campaigns. The greatest bias occurred in the scenario in which the direction of misclassification differed by case or control status. The smallest vaccine effectiveness estimates were observed when unvaccinated cases were misclassified as vaccinated, and vaccinated controls were misclassified as unvaccinated. Conversely, the largest vaccine effectiveness estimates occurred when vaccinated cases were misclassified as unvaccinated and unvaccinated controls were misclassified as vaccinated. An advantage with the test-negative design compared with other observational studies is that vaccination status is ascertained at the same timepoint (when health care is sought) for both cases and controls, ideally before their test status is known. This strategy reduces the potential for differential misclassification compared with a design in which cases and controls are recruited from separate locations with differential access to vaccination records.

Randomised controlled trials have previously been used to evaluate the test-negative design for influenza vaccines, rotavirus, and enterovirus-71 vaccines, and for respiratory syncytial virus monoclonal antibody.^19^, ^23^, ^29^ We did not identify any published evaluations of the test-negative design for typhoid vaccines in endemic countries, although one study used a modified test-negative design to evaluate effectiveness of a Vi*-*polysaccharide vaccine in travellers. In that study, participants with blood cultures positive for *S enterica* serovar Paratyphi were used as controls and the core assumption of the test-negative design was not examined.^30^ Our analyses support that Vi-TT has no effect on non-typhoid fever and show that the test-negative design is a robust method to efficiently estimate vaccine effectiveness in settings where 10% or fewer blood culture specimens are positive for *S* Typhi, and where vaccination status can be reliably ascertained. These are important findings as more LMICs prepare to introduce typhoid conjugate vaccine, and as new typhoid conjugate vaccines for which efficacy estimates from randomised trials are not available become approved for use. Furthermore, vaccine effectiveness estimated by the test-negative design can be used to build upon randomised trial-established vaccine efficacy estimates by measuring longer-term effectiveness of the recommended single-dose typhoid conjugate vaccine over time, as current trials were limited to a maximum of 4 years of follow-up.^4^, ^5^, ^6^ Strategies to accurately document past vaccination exposure are particularly crucial as the time interval between vaccination and outcome ascertainment increases.

In conclusion, post-introduction vaccine evaluations are a crucial component of programme monitoring. Such evaluations can affect other countries’ decisions to introduce vaccines and can play a part in maintaining continued support in the countries of introduction. The test-negative design expands the options for post-introduction typhoid conjugate vaccine evaluation and is well-suited for low-income settings due to its efficiency, convenience, and low cost. To our knowledge, this study is the first to rigorously evaluate the utility of the test-negative design to estimate typhoid conjugate vaccine effectiveness using a large randomised trial in Malawian children. Future evaluations of the test-negative design with different typhoid conjugate vaccines, vaccination coverage rates, or follow-up periods in different countries are needed to confirm the study findings in different populations and settings.

## Data sharing

Data will be shared in accordance with the Bill & Melinda Gates Foundation's policy. De-identified individual participant data, including data dictionaries from this study, will be made available to researchers without restriction when the study, analysis, and reporting are complete. Data can be obtained by contacting TyVAC@som.umaryland.edu.

## Declaration of interests

YL, AJD, KMN, SD, MBL, and LPJ receive salary from the Typhoid Vaccine Acceleration Consortium (TyVAC) grant from the Bill & Melinda Gates Foundation. KMN is a member of the WHO Strategic Advisory Group of Experts on Immunization. MV reports grants from the Bill & Melinda Gates Foundation, National Institute for Health Research, and Medical Research Council. NF reports grants from the Bill & Melinda Gates Foundation and Wellcome and serves as a steering group member of a Gates-funded study of pneumococcal vaccine schedules in The Gambia. All other authors declare no competing interests.
