# Supplementary material for: Typhoid conjugate vaccine effectiveness in Malawi: evaluation of a test-negative design using randomised, controlled clinical trial data
Source: Lancet Glob Health. 2022 Nov 25;11(1):e136–44. doi: 10.1016/S2214-109X(22)00466-1 (PMC9748309; doi:10.1016/S2214-109X(22)00466-1)
Supplement: Supplementary appendix [file mmc1.pdf]

THE LANCET
Global Health

Supplementary appendix

This appendix formed part of the original submission and has been peer reviewed. We post it as supplied by the authors.

Supplement to: Liang Y, Driscoll AJ, Patel PD, et al. Typhoid conjugate vaccine effectiveness in Malawi: evaluation of a test-negative design using randomised, controlled clinical trial data. *Lancet Glob Health* 2022; published online Nov 25. https://doi.org/10.1016/S2214-109X(22)00466-1.

Supplementary Materials

Section A: Mahalanobis multivariate-distance nearest-neighbor matching

Mahalanobis multivariate-distance nearest-neighbor matching without replacement is an effective tool that we used to generate a 1:3 case-control matched sample. Before matching, there were 101 test-positive specimens (cases) and 8,060 test-negative specimens (controls). For each case, we wanted to select three controls matched on age categories (<2 vs. 2-<5 vs. ⩾ 5), study site (Ndirande vs. Zingwangwa), and the date of blood culture (BC). We used the "kmatch" function in Stata/SE for the multivariate-distance matching, which uses Mahalanobis matching by default instead of other methods such as Euclidean, on the above three variables simultaneously, with exact matches on the age and study site dummy variables so that each case and its selected three controls were in the same age category and from the same study site. Without replacement matching was used so that no controls can be matched to multiple cases, resulting in 303 (=101 cases × 3) unique controls. The balancing diagnostics (see the tables and the plot below) indicate that the matching was very successful on both means (StdDif=0) and variances (Ratio=1). In addition, BC dates were matched within 20 days for each case-control pair (97·4% were matched within 7 days).

	Before Matching			After Matching		
**Means**	**Cases**	**Controls**	**StdDif**	**Cases**	**Controls**	**StdDif**
**age_cat1**	.148515	.247643	-.250152	.148515	.148515	0
**age_cat2**	.207921	.346402	-.312489	.207921	.207921	0
**Zingwangw**	.50495	.420347	.16986	.50495	.50495	0
**date_BC**	21874.7	21749	.371624	21874.7	21874.8	-.00039

	Before Matching			After Matching		
**Variances**	**Cases**	**Controls**	**Ratio**	**Cases**	**Controls**	**Ratio**
**age_cat1**	.1277228	.1863389	.6854327	.1277228	.1268769	1.006667
**age_cat2**	.1663366	.2264357	.7345865	.1663366	.1652351	1.006667
**Zingwangw**	.2524752	.2436857	1.036069	.2524752	.2508032	1.006667
**date_BC**	127312.2	101497.9	1.254333	127312.2	126522.4	1.006242

Age_cat1=1 if age<2; 0 otherwise
Age_cat2=1 if age>=2 and age<5; 0 otherwise
Zingwangw=1 if study site = Zingwangw; 0 if study site = Ndirande
date_BC: date of blood culture. In Stata, a date variable is the number of days from Jan. 1, 1960. For example, date_BC=0 means Jan. 1, 1960; date_BC=21874 means Nov. 21, 2019.
StdDif: standardized difference

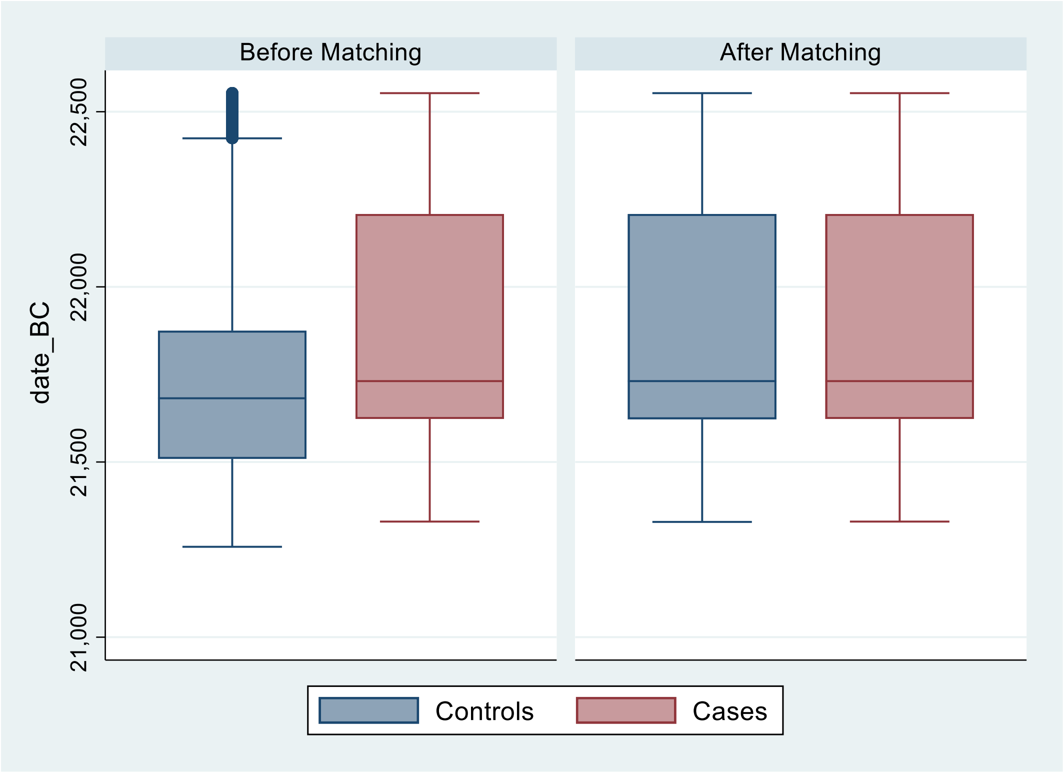

Reference:

Jann B. KMATCH: Stata module module for multivariate-distance and propensity-score matching, including entropy balancing, inverse probability weighting, (coarsened) exact matching, and regression adjustment. Statistical Software Components S458346: Boston College Department of Economics; 2017.

Section B: Vaccine misclassification analysis in Table 3

Table 3: Effect of vaccine misclassification on vaccine effectiveness estimation by test-negative design specimen-based analysis[a]

Overall vaccine misclassification rate (p_1+p_2)	Scenario	% cases vaccinated by Vi-TT	% controls vaccinated by Vi-TT	VE against typhoid[b] [95% CI]
0%	Gold standard	17/101 (16·8%)	4092/8060 (50·8%)	80·4% [66·9%, 88·4%]
5%	Misclassifying vaccinated as unvaccinated, both groups[c]	12/101 (11·9%)	3689/8060 (45·8%)	84·0% [70·8%, 91·3%]
	Differential misclassification, lowest possible VE[d]	22/101 (21·8%)	3689/8060 (45·8%)	67·0% [47·0%, 79·5%]
	Differential misclassification, highest possible VE[e]	12/101 (11·9%)	4495/8060 (55·8%)	89·3% [80·4%, 94·2%]

p_1=Probability of misclassifying vaccinated as unvaccinated; p_2=Probability of misclassifying unvaccinated as vaccinated
VE = vaccine effectiveness; CI = confidence interval; OR = odds ratio
a. See supplementary Section B for additional details
b.VE=(1-OR)×100%
c. Only misclassifying vaccinated as unvaccinated for both cases and controls due to the loss of vaccination cards, that is, $p_1+p_2=p_1$, hence p_2=0 among both groups
d. p_1=0 among cases (misclassifying unvaccinated as vaccinated among cases) and p_2=0 among controls (misclassifying vaccinated as unvaccinated among controls), resulting in the lowest possible VE
e. p_2=0 among cases (misclassifying vaccinated as unvaccinated among cases) and p_1=0 among controls (misclassifying unvaccinated as vaccinated among controls), resulting in the highest possible VE

Please review the results above as an example. If there was no vaccine misclassification, as occurs in an RCT, 17 out of 101 (16.8%) cases were vaccinated and 4,092 out of 8,060 (50.8%) controls were vaccinated, resulting in a VE of 80.4%. If the overall vaccine misclassification rate was 5% and both cases and controls were equally likely to be misclassified (since we expect that poor vaccination records affect both cases and controls equally), a total of (101+8060)×5%=408 specimens (5 cases vs. 403 controls) would have a vaccination status that is misclassified. If the probability of misclassifying vaccinated as unvaccinated (p_1) is the same as the probability of misclassifying unvaccinated as vaccinated (p_2), for example, among the 5 vaccination-status misclassified cases, 2 vaccinated were misclassified as unvaccinated and 3 unvaccinated were misclassified as vaccinated, then 17-2+3=18 cases were counted as vaccinated, which was close to the true value of 17. Similarly, among the 403 vaccination-status misclassified controls, if 201 vaccinated were misclassified as unvaccinated and 202 unvaccinated were misclassified as vaccinated, then 4092-201+202=4093 controls were counted as vaccinated, which was also close to the true value of 4092. Hence, on average, the expected value of VE will be unchanged if the probability of misclassifying vaccinated as unvaccinated is the same as the probability of misclassifying unvaccinated as vaccinated (i.e., $p_1=p_2$).

If p_2=0 among both cases and controls, then $p_1+p_2=p_1$=5%. That is, among the 5 vaccination-status misclassified cases and the 403 vaccination-status misclassified controls, misclassification occurred in one direction only: vaccinated were misclassified as unvaccinated due to the loss of vaccination cards. In this scenario, 17-5=12 cases were counted as vaccinated and 4092-403=3689 controls were counted as vaccinated, resulting in a VE of 84%.

If differential misclassification can occur between cases and controls, the vaccination rate among cases would be highest when 5 unvaccinated cases were misclassified as vaccinated; and the vaccination rate among controls would be lowest when 403 vaccinated controls were misclassified as unvaccinated. In this scenario, 17+5=22 cases were counted as vaccinated and 4092-403=3689 controls were counted as vaccinated, resulting in the smallest difference in vaccination rate between the cases and the controls, hence the lowest possible VE of 67%. On the other hand, the vaccination rate among cases would be lowest when 5 vaccinated cases were misclassified as unvaccinated; and the vaccination rate among controls would be highest when 403 unvaccinated controls were

misclassified as vaccinated. In this scenario, 17-5=12 cases were counted as vaccinated and 4092+403=4495 controls were counted as vaccinated, resulting in the largest difference in vaccination rate between the cases and the controls, hence the highest possible VE of 89.3%.

The same logic applies to other vaccine misclassification rates displayed in Table 3.

Section C: Blood culture (BC) positivity rate and BC sensitivity analyses in Table 4

Table 4: Effect of blood culture positivity rate and blood culture test sensitivity on vaccine effectiveness estimation by test-negative design specimen-based analysis[a]

Observed % BC typhoid positive	BC sensitivity	Adjusted % BC typhoid positive[b]	Adjusted % cases vaccinated[b]	Adjusted % controls vaccinated[b]	Adjusted VE against typhoid[b,c] [95% CI]
**101/8161 (1·2%)**	**100%**	**101/8161 (1·2%)**	**17/101 (16·8%)**	**4092/8060 (50·8%)**	**80·4% [66·9%, 88·4%]**
	80%	126/8161 (1·5%)	21/126 (16·7%)	4088/8035 (50·9%)	80·7% [69·1%, 87·9%]

Please review the results above as an example. In our TND specimen-based sample, there were 101 typhoid positive specimens (cases) from a total of 8,161 specimens, resulting in a BC positivity rate of 1.2%. Let P be the number of real positive cases. If the BC sensitivity is 100%, no adjustment is needed; hence the adjusted BC positivity rate is still 101/8161=1.2%. If the BC sensitivity is 80%, then 101/P=0.8, hence P=101/0.8=126 and the adjusted BC positivity rate should be P/8161=126/8161=1.5%. In this scenario, there should be 126 real positive cases (instead of 101 cases before adjustment) and 8161-126=8035 real negative controls (instead of 8060 controls before adjustment). We assume that there was no vaccine misclassification, hence a total of 17+4092=4109 specimens were from vaccinated children. In addition, we assume that the vaccination rate among the 126-101=15 missed cases (i.e., false negatives) was the same as the vaccination rate among the 101 observed cases (i.e., true positives). Therefore, among the 126 real positive cases, 126×17/101=21 were vaccinated; and, among the 8035 real negative controls, 4109-21=4088 were vaccinated, resulting in a VE of 80.7%. The same logic applies to other sensitivity values displayed in Table 4.

Table 4: Effect of blood culture positivity rate and blood culture test sensitivity on vaccine effectiveness estimation by test-negative design specimen-based analysis[a]

Observed % BC typhoid positive	BC sensitivity	Adjusted % BC typhoid positive[b]	Adjusted % cases vaccinated[b]	Adjusted % controls vaccinated[b]	Adjusted VE against typhoid[b,c] [95% CI]
**101/8161 (1·2%)**	**100%**	**101/8161 (1·2%)**	**17/101 (16·8%)**	**4092/8060 (50·8%)**	**80·4% [66·9%, 88·4%]**
**408/8161 (5·0%)**	**100%**	**408/8161 (5·0%)**	**69/408 (16·9%)**	**4040/7753 (52·1%)**	**81·3% [75·7%, 85·6%]**

If we assume the BC positivity rate should be 5% instead of 1.2%, then 8161×5%=408 should be real positive cases and 8161-408=7753 should be real negative controls, and the total number of vaccinated is still 4109 due to the no vaccine misclassification assumption. Among the 408 real positive cases, 408×17/101=69 are vaccinated; and, among the 7753 real negative controls, 4109-69=4040 are vaccinated, resulting in a VE of 81.3%. The same logic applies to other observed BC positivity values displayed in Table 4.

Table S1: Subgroup analyses by age, sex, and study site

	Total	Participant-based analysis			No BC	Specimen-based analysis[c]	
		Test-positive for typhoid, n (column %)	Test-negative no censoring[a], n (column %)	Test-negative with censoring[b], n (column %)		Test-positive specimens, n (column %)	Test-negative specimens, n (column %)
**Age < 5**							
Vi-TT	5,044	8 (23·5)	1,704 (49·7)	1,699 (49·8)	3,337	9 (25·0)	2,412 (50·4)
MenA	5,158	26 (76·5)	1,727 (50·3)	1,710 (50·2)	3,422	27 (75·0)	2,376 (49·6)
Total	10,202	34	3,431	3,409	6,759	36	4,788
VE against typhoid[d] [95% CI], p value	··	68·6% [30·6%, 85·8%][e] 0·004	68·8% [30·9%, 85·9%][f] 0·002	69·0% [31·4%, 86·0%][g] 0·002	··	··	67·2% [30·0%, 84·6%][h] 0·002
VE against non-typhoid[i] [95% CI], p value	··	··	-1·1% [-6·8%, 4·2%][f] 0·69	-1·6% [-7·3%, 3·8%][g] 0·60	··	··	-2·7% [-7·3%, 1·7%][h] 0·23
**Age >= 5**							
Vi-TT	8,901	8 (12·7)	1,383 (50·6)	1,378 (50·8)	7,515	8 (12·3)	1,680 (51·3)
MenA	8,779	55 (87·3)	1,352 (49·4)	1,334 (49·2)	7,390	57 (87·7)	1,592 (48·7)
Total	17,680	63	2,735	2,712	14,905	65	3,272
VE against typhoid[d] [95% CI], p value	··	85·8% [70·1%, 93·2%][e] <0·001	85·8% [70·0%, 93·2%][f] <0·001	85·9% [70·3%, 93·3%][g] <0·001	··	··	86·7% [72·0%, 93·7%][h] <0·001
VE against non-typhoid[i] [95% CI], p value	··	··	-1·0% [-8·2%, 5·7%][f] 0·78	-1·9% [-9·2%, 4·9%][g] 0·60	··	··	-3·6% [-10·3%, 2·6%][h] 0·26
**Male**							
Vi-TT	6,925	8 (16·7)	1,528 (50·6)	1,524 (50·8)	5,393	9 (17·7)	2,050 (51·4)
MenA	6,759	40 (83·3)	1,492 (49·4)	1,477 (49·2)	5,242	42 (82·3)	1,940 (48·6)
Total	13,684	48	3,020	3,001	10,635	51	3,990
VE against typhoid[d] [95% CI], p value	··	80·6% [58·5%, 90·9%][e] <0·001	80·5% [58·1%, 90·9%][f] <0·001	80·6% [58·5%, 91·0%][g] <0·001	··	··	79·7% [58·2%, 90·2%][h] <0·001
VE against non-typhoid[i] [95% CI], p value	··	··	-0.1% [-6·6%, 6·0%][f] 0·97	-0.7% [-7·3%, 5·5%][g] 0·83	··	··	-2·4% [-8·0%, 2·8%][h] 0·37
**Female**							
Vi-TT	7,020	8 (16·3)	1,559 (49·6)	1,553 (49·8)	5,459	8 (16·0)	2,042 (50·2)
MenA	7,178	41 (83·7)	1,587 (50·4)	1,567 (50·2)	5,570	42 (84·0)	2,028 (49·8)
Total	14,198	49	3,146	3,120	11,029	50	4,070
VE against typhoid[d] [95% CI], p value	··	80·2% [57·8%, 90·7%][e] <0·001	80·1% [57·5%, 90·7%][f] <0·001	80·3% [57·9%, 90·8%][g] <0·001	··	··	81·1% [59·6%, 91·1%][h] <0·001
VE against non-typhoid[i] [95% CI], p value	··	··	-0·6% [-7·0%, 5·4%][f] 0·84	-1·3% [-7·8%, 4·8%][g] 0·67	··	··	-2·4% [-8·0%, 2·8%][h] 0·37

Table S1: Subgroup analyses by age, sex, and study site (cont.)

	Participant-based analysis					Specimen-based analysis[c]	
	Total	Test-positive for typhoid, n (column %)	Test-negative no censoring[a], n (column %)	Test-negative with censoring[b], n (column %)	No BC	Test-positive specimens, n (column %)	Test-negative specimens, n (column %)
**Ndirande**							
Vi-TT	8,772	9 (18·8)	1,858 (50·1)	1,852 (50·2)	6,911	10 (20·0)	2,365 (50·6)
MenA	8,738	39 (81·2)	1,853 (49·9)	1,839 (49·8)	6,860	40 (80·0)	2,307 (49·4)
Total	17,510	48	3,711	3,691	13,771	50	4,672
VE against typhoid[d] [95% CI], p value	··	77·1% [52·8%, 88·9%][e] <0·001	77·0% [52·4%, 88·9%][f] <0·001	77·1% [52·6%, 88·9%][g] <0·001	··	··	75·6% [51·1%, 87·8%][h] <0·001
VE against non-typhoid[i] [95% CI], p value	··	··	-0% [-5·9%, 5·6%][f] 0·99	-0·3% [-6·2%, 5·3%][g] 0·91	··	··	-1·6% [-6·8%, 3·3%][h] 0·52
**Zingwangwa**							
Vi-TT	5,173	7 (14·3)	1,229 (50·1)	1,225 (50·4)	3,941	7 (13·7)	1,727 (51·0)
MenA	5,199	42 (85·7)	1,226 (49·9)	1,205 (49·6)	3,952	44 (86·3)	1,661 (49·0)
Total	10,372	49	2,455	2,430	7,893	51	3,388
VE against typhoid[d] [95% CI], p value	··	83·4% [63·0%, 92·5%][e] <0·001	83·4% [62·8%, 92·6%][f] <0·001	83·6% [63·4%, 92·7%][g] <0·001	··	··	84·7% [65·9%, 93·1%][h] <0·001
VE against non-typhoid[i] [95% CI], p value	··	··	-1·1% [-8·3%, 5·7%][f] 0·76	-2·2% [-9·5%, 4·7%][g] 0·55	··	··	-3·6% [-9·7%, 2·0%][h] 0·21

a.Controls include participants with an episode of non-typhoid illness, without censoring for typhoid (i.e., controls may have tested positive for typhoid at another time point)

b. Controls include participants with an episode of non-typhoid illness, with censoring for typhoid (i.e., controls exclude participants who ever had a test that was typhoid positive during the study period)

c. Cases are typhoid positive specimens and control are typhoid negative specimens

d. VE=(1-OR)×100% using the TND sample only

e. VE=(1- IRR)×100%

f. TND method A

g. TND method B

h. TND method C

i. VE=(1-RR)×100% using the whole RCT

BC = blood culture; MenA = meningococcal capsular group A conjugate vaccine; Vi-TT = Vi polysaccharide typhoid conjugate vaccine;
RCT = randomized controlled trial; TND = test-negative design; VE = vaccine efficacy in RCT or vaccine effectiveness in TND;
CI = confidence interval; IRR = incidence rate ratio; RR = risk ratio; OR = odds ratio

Table S2: Summary of vaccine effectiveness against typhoid estimates using different study designs

Study design	VE method	VE against typhoid [95% CI]
RCT	(1-IRR)×100%	80·4% [66·4%, 88·5%]
	(1-RR)×100%	80·3% [66·3%, 88·4%]
TND specimen-based, all controls used (101 cases vs. 8,060 controls)	(1-OR)×100%	80·4% [66·9%, 88·4%]
TND specimen-based **1:3 case-control matched**, exact matching on age groups (<2, 2-<5, >=5) and study site, BC date matched within 20 days (97.4% matched within 7 days)[a]	(1-OR)×100% unadjusted for matching	80·9% [66·4%, 89·2%]
	(1-OR)×100% adjusted for matching using mixed-effects logistic regression	80·9% [66·4%, 89·2%]

a. Mahalanobis multivariate-distance nearest-neighbor matching without replacement (101 cases vs. 303 controls)
VE = vaccine efficacy or vaccine effectiveness; RCT = randomized controlled trial
IRR = incidence rate ratio; RR = risk ratio; OR = odds ratio; CI = confidence interval
